# Vibration thresholds in carpal tunnel syndrome assessed by multiple frequency vibrometry: a case-control study

**DOI:** 10.1186/s12995-017-0181-6

**Published:** 2017-12-08

**Authors:** Magnus Flondell, Birgitta Rosén, Gert Andersson, Tommy Schyman, Lars B. Dahlin, Anders Björkman

**Affiliations:** 1 Department of Hand Surgery, Skåne University Hospital, Jan Waldenströms gata 5, 20502 Malmö, SE Sweden; 2 Departments of Neurophysiology, Skåne University Hospital, Malmö, Sweden; 3 Department of Clinical Studies Sweden – Forum South, Skåne University Hospital, Malmö, Sweden; 4 Department of Translational Medicine – Hand Surgery, Lund University, Malmö, Sweden; 5 Department of Clinical Sciences, Lund University, Lund, Sweden

**Keywords:** Carpal tunnel syndrome, Vibrometry, Sensibility, Touch thresholds, Vibrotactile sense, Vibration perception threshold

## Abstract

**Background:**

Carpal tunnel syndrome (CTS) is the most common compression neuropathy, but there is no gold standard for establishing the diagnosis. The ability to feel vibrations in the fingertips is dependent on the function in cutaneous receptors and afferent nerves. Our aim was to investigate vibration perception thresholds (VPTs) in patients with CTS using multi-frequency vibrometry.

**Methods:**

Sixty-six patients (16 men and 50 women) with CTS, diagnosed from clinical signs and by electroneurography, and 66 matched healthy controls were investigated with multi-frequency vibrometry. The VPTs were assessed at seven frequencies (8, 16, 32, 64, 125, 250, and 500 Hz) in the index finger and little finger bilaterally. The severity of the CTS was graded according to Padua and the patient’s subjective symptoms were graded according to the Boston carpal tunnel questionnaire. Touch thresholds were assessed using the Semmes-Weinstein monofilaments.

**Results:**

Patients with CTS had significantly higher VPTs at all frequencies in the index finger and in 6 out of 7 frequencies in the little finger compared to the controls. However, the VPT was not worse in patients with more severe CTS. Patients with unilateral CTS showed significantly higher VPTs in the affected hand. There were no correlations between VPTs and electrophysiological parameters, subjective symptoms, or touch threshold.

**Conclusions:**

Patients with CTS had impaired VPTs at all frequencies compared to the controls. Since the VPTs are dependent on function in peripheral receptors and their afferent nerves, multi-frequency vibrometry could possibly lead to diagnosis of CTS.

## Background

Carpal tunnel syndrome (CTS), where the median nerve is compressed in the carpal tunnel, is the most common compression neuropathy [1] with a prevalence in the general population of 2.7–5.8% [2]. CTS is known to affect both large- and small-diameter myelinated nerve fibers and also unmyelinated nerve fibers [3]. There is no “gold standard” for the diagnosis of CTS, which is generally based on a medical history of sensory disturbances and pain in median nerve-innervated fingers [4] in combination with positive clinical tests. In patients with atypical symptoms and signs, an electroneurography (ENeG) is often used to support the diagnosis. However, there is considerable controversy regarding the need for ENeG in patients with suspected CTS. Some consider ENeG to be mandatory before planning surgery for CTS [5], while others question the need for ENeG [6]. Frequently quoted reasons for not using ENeG in the diagnostic work-up for suspected CTS include treatment delay, inconvenience, patient discomfort, costs, the need for specialist competence to perform and interpret the investigation, and poor correlation between ENeG and clinical symptoms [7, 8].

The ability to feel vibrations, i.e. vibrotactile sense, is dependent on the function in cutaneous receptors and large-diameter (Aβ) afferent nerves. The vibration perception threshold (VPT), which is the lowest intensity that can be perceived at a particular frequency, is known to be impaired early in different neuropathies [9–11]. Because of this, various methods have been proposed to analyze the VPTs. The VPT can be assessed at a single frequency using a tuning fork (128 Hz) or by vibrometry (100–120 Hz). A technique, multi-frequency vibrometry, whereby the VPT is assessed at seven different frequencies has been shown to increase the sensitivity of detecting neuropathies compared to when a single frequency is used [9, 12, 13].

Altered VPTs at different frequencies may reflect dysfunction of different mechanoreceptors, such as the Pacinian corpuscles (sensitive at a maximum of 250 Hz), the Meissner corpuscles (5–50 Hz), and the Merkel corpuscles (< 15 Hz) and their associated Aβ-sensory fibers [12]. Thus, assessment of VPTs at multiple frequencies has been suggested as a diagnostic tool in diabetic neuropathy and in patients with neuropathy due to long-term exposure to hand-held vibrating tools [14, 15]. Because function in Aβ-sensory fibers is also affected early in compression neuropathies, evaluation of VPTs at different frequencies may be useful in the diagnostic work-up of patients with suspected CTS [14]. Our aim was to investigate VPTs using multi-frequency vibrometry in patients with idiopathic CTS.

## Methods

### Patients

Over a 6-year period (2009–2015) patients who were referred to the Department of Hand Surgery, Malmö, Sweden with suspected CTS were screened by a hand surgeon for participation in the study.

The inclusion criteria were as follows: subjective symptoms of CTS for more than 3 months, classic or probable CTS according to Katz’ hand diagram [4, 16], clinical signs of CTS with a positive Tinels and Phalens test, age between 18 and 70 years, and an ENeG with a fractionated sensory nerve conduction velocity for the median nerve across the wrist of 40 m/s or less. Exclusion criteria were: having been operated for CTS previously, prior wrist or carpal fracture, diabetes, thyroid disease, rheumatoid arthritis, neurological disease, drug abuse, complete conduction block on ENeG or previous regular exposure to hand-held vibrating tools. Participants had to be able to read and understand Swedish in order to be able to fill out the patient-rated outcome measures (PROMs) in the proper way.

For each patient, a similar-aged (within 5 years), gender-matched, and handedness-matched healthy control was identified from a population study cohort collected at our clinic. The same exclusion criteria as used for the study patients were applied. Furthermore, the controls were not to have any sensory deficiencies, pain in the hands, or previous neuropathies.

### Assessments

Patients were subjected to a clinical an electrophysiological examination in order to support the diagnosis of CTS. Furthermore, patients and controls were examined with multi-frequency vibrometry.

#### Clinical assessment

A specialist in hand surgery did all clinical examinations and secured the diagnosis. Subjective symptoms were assesses using Katz’ hand diagram [4, 16]. A clinical evaluation was performed where the patients were assessed for weakness in thumb abduction and for signs of atrophy in the thenar muscles. Furthermore Tinels test was performed on the median nerve just proximal to the flexor retinaculum, the test was considered positive if the patient experienced tingling in median nerve innervated fingers during the test [17]. Phalens test for carpal tunnel syndrome was performed as well. The test was considered positive if the patient experienced tingling in median nerve innervated fingers within one minute after commencing the test [17].

#### Electrophysiology

A standard electrophysiological assessment (ENeG) was performed on both arms. Orthodromic sensory ENeG was performed by stimulating the thumb, the index finger and the long finger for the median nerve and the little finger for assessment of the ulnar nerve. The stimulation ring electrodes were placed at the proximal interphalangeal and distal interphalangeal joints for the index, long, and little finger and just proximal and distal to the interphalangeal joint of the thumb. Recording electrodes were placed over the respective nerves at the proximal wrist crease and three cm more proximal. Fractionated, antidromic sensory neurography was performed on the median nerve with recording ring electrodes placed over the proximal and distal interphalangeal joints of the long finger. The stimulation sites were in the palm, at the proximal wrist crease and proximal to the elbow. The nerve conduction velocity in the segment between the wrist crease and the palm was calculated.

For motor conduction studies, recordings were performed from the abductor pollicis brevis muscle (innervated by the median nerve) and abductor digiti minimi muscle (innervated by the ulnar nerve) with stimulation of the respective nerves 80 mm proximal to the electrode placed over the muscle. The patient’s skin temperature was over 30 °C during the ENeG. The ENeG included sensory conduction velocity (SCV), sensory nerve action potential (SNAP), and distal motor latency (DML), and was performed on a Nicolet Viking Select Electromyograph (Nicolet Brand Products, Middleton, WI, USA). All examinations were performed by the same technician and were evaluated independently by the same neurophysiologist.

Based on the results from ENeG, the severity of each patient’s CTS was classified according to Padua et al. [18]. However, one inclusion criteria was a fractionated sensory nerve conduction velocity for the median nerve across the wrist of 40 m/s or less. Furthermore, patients with a complete conduction block were excluded. This means that only patients graded as mild and moderate CTS, according to Padua, were eligible for the study.

#### Multi-frequency vibrometry

Vibration perception thresholds were measured at multiple frequencies using a VibroSense Meter® (Vibrosense Dynamics, Malmö, Sweden) in accordance with previously described technique [19]. Before the examination, the operator explained the examination procedure to the subject. Essentially, the median and ulnar nerves were evaluated by recording bilateral vibration thresholds at the finger pulps of the index finger (innervated by median nerve) and little finger (innervated by the ulnar nerve) bilaterally. The patients wore acoustic ear-muffs to avoid bias from sound emitted by the vibration pin of the measurement unit. Since sensibility varies with temperature [20], the finger temperature was monitored and had to be above 30 °C before assessment. The patient placed the finger to be examined on the vibration pin. When the patient perceived vibration, he or she indicated this by pressing a switch and by holding it depressed until vibrations were no longer felt. The VibroSense Meter® administers vibration at seven different frequencies (8, 16, 32, 64, 125, 250, and 500 Hz), and a median threshold value, expressed in decibel (dB), was recorded for each frequency from the index and little fingers of both hands. The examination, index and little fingers of both hands, took 20 min to complete. The patients and controls were examined in the same way, except that the controls were only assessed in the dominant index finger and little finger. All examinations were performed by one out of two technician who had more than 5-years of experience in doing multifrequency vibrometry.

#### Assessment of touch threshold

Assessment of cutaneous touch/pressure thresholds was done on the tip of the index and little fingers on both hands in patients with CTS, and on the index finger of the dominant hand in controls, using a set of 20 Semmes-Weinstein monofilaments (SWM) (North Coast Medical Inc., Gilroy, CA, USA). Assessment was started with SWM no. 2.83 (representing a pressure of 70 mg) and thereafter continued in an ascending or descending order depending on the answer for the first filament. Each filament was applied three times according to a standardized procedure [21]. Results were quantified from 0 to 20, representing the 20 monofilaments, with 20 corresponding to the lowest threshold. To familiarize the test subject with the test procedure and to eliminate the possibility of biased results due to learning effects, testing for touch threshold on the third digit was performed before testing the study fingers.

#### Patient-rated outcome measures (PROMs)

Symptom severity score (SSS) from the Boston questionnaire [22], which assess subjective severity of symptom in patients with CTS, was recorded at the time of inclusion.

### Statistical analysis

IBM SPSS Statistics (Statistical Package for the Social Sciences, version 23 for Mac; IBM Corp., Armonk, NY, USA) was used for the statistical assessment of data. Values are presented as median and interquartile range (IQR).

As this was a matched-control study, there is no need to adjust for confounding variables when comparing cases and controls. Due to this, the Wilcoxon signed-rank test was used to evaluate any statistical difference between patients with CTS and the control group. The same method was used to evaluate whether there was any difference between hands in the same patient. When comparing subgroups according to Padua, the Mann-Whitney U-test was used for continuous and nominal variables. Fisher’s exact test was used for categorical variables. Spearman’s correlation for non-parametric testing was used for investigation of correlations. Any *p*-value less than 0.05 were considered significant.

## Results

### Demographics

Informed consent was obtained from 66 patients (16 men and 50 women, median age 50 years, IQR 13) (Table 1) and 66 age- and gender-matched healthy controls (median age 46.5, IQR 11). Of the 66 patients, 11 were rated as having mild CTS (abnormal SCV and normal DML) and 55 having moderate CTS (abnormal SCV and abnormal DML) according to Padua [18] (Table 1). Thirty-eight patients had unilateral CTS.

**Table 1:** Characteristics of patients, in total and sub-divided based on severity according to Padua

CTS grade	Mild (*n* = 11)	Moderate (*n* = 55)	Total (*n* = 66)	*P*-value
Age, years	46[15]	50[13]	50[13]	0.67
Sex, F/M	11/0	39/16	50/16	*0.05*
Bilat./unilat.	2/9	26/29	28/38	0.10
ENeG SCV, m/s	36[7]	30[6]	31[6]	*0.001*
ENeG SNAP, μV	12[9]	5[5]	6[7]	*0.03*
ENeG DML, ms	3.9[0.3]	5.3[1.0]	5.0[1.2]	*< 0.001*
SSS, score	2.72[1.4]	2.45[0.78]	2.55[0.73]	0.34
SWM, no.of filament	18[1]	18[2]	18[2]	0.96

Data are median [IQR]. *P*-values are based on Mann-Whitney U-test for continuous and nominal data and on Fisher’s exact test for categorical variables

*SCV* sensory conduction velocity, *SNAP* sensory nerve action potential, *DML* distal motor latency, *SSS* symptom severity score, *SWM* Semmes-Weinstein monofilament

Values in italics represent statistically significant differences

There were significantly more women in the group with mild CTS. Age, PROMs and touch thresholds were not significantly different between the Padua groups.

### Multi-frequency vibration perception thresholds

The VPTs were significantly higher, indicating poorer capability to detect vibrations, at all frequencies in the index finger in patients with CTS than in the healthy controls (Table 2). In addition, VPTs in the little finger were significantly higher at all frequencies in patients with CTS than in the controls, except at the highest frequency (500 Hz) (Table 2). When we compared patients who were classified as having mild CTS with their individual controls, the patients had significantly higher VPTs in the index finger at all frequencies except at 500 Hz. Patients with moderate CTS had significantly higher VPTs at all frequencies (Table 3). However, when we compared VPTs in the index finger between patients who were classified as having mild and moderate CTS, there were no significant differences.

**Table 2:** Vibration perception thresholds in patients with carpal tunnel syndrome (CTS) and in healthy controls

Index finger	8 Hz	16 Hz	32 Hz	64 Hz	125 Hz	250 Hz	500 Hz
CTS (*n* = 66)	108.6[4.9]	115.9[5.1]	117.5[7.8]	109.0[11.7]	110.3[10.3]	120.0[11.3]	133.4[13.1]
Controls (*n* = 66)	105.0[6.6]	112.5[7.7]	113.1[6.1]	103.1[7.0]	102.0[9.7]	110.1[12.7]	125.9[13.2]
*P*-value	*< 0.001*	*0.001*	*< 0.001*	*< 0.001*	*< 0.001*	*< 0.001*	*< 0.001*
Little finger	8 Hz	16 Hz	32 Hz	64 Hz	125 Hz	250 Hz	500 Hz
CTS (*n* = 66)	106.9[7.1]	115.1[7.0]	120.1[7.2]	111.2[10.1]	108.6[9.5]	115.4[14.5]	127.6[14.8]
Controls (*n* = 66)	105.0[5.1]	111.9[4.7]	115.4[10.1]	107.4[9.8]	104.1[11.2]	111.0[10.0]	125.9[16.2]
*P*-value	*0.009*	*0.005*	*< 0.001*	*< 0.001*	*0.001*	*0.005*	0.17

Vibration perception thresholds are expressed in dB. Data are median [IQR]. P-values are based on Wilcoxon’s signed-rank test

Values in italics represent statistically significant differences

**Table 3:** Vibration perception thresholds in patients with carpal tunnel syndrome (CTS) sub-divided according to Padua

Index finger	8 Hz	16 Hz	32 Hz	64 Hz	125 Hz	250 Hz	500 Hz
Mild CTS (*n* = 11)	108.5[4.5]	116.2[10.0]	119.1[9.1]	109.0[11.9]	109.8[8.2]	117.8[10.2]	132.4[9.5]
Controls (*n* = 11)	99.4[6.3]	108.2[8.5]	107.8[7.3]	101.0[3.9]	97.2[9.6]	108.3[14.3]	125.3[17.1]
*P*-value	*0.011*	*0.041*	*0.006*	*0.008*	*0.016*	*0.050*	0.062
Moderate CTS (*n* = 55)	108.9[5.3]	115.6 [4.3]	117.3[7.8]	109.0[11.6]	110.5[10.7]	120.4[12.3]	133.9[15.4]
Controls (*n* = 55)	105.8[6.0]	113.3 [7.5]	113.6[5.0]	104.9[7.4]	102.8[9.8]	110.2[11.3]	125.9[12.7]
*P*-values	*0.009*	*0.010*	*0.001*	*0.001*	*< 0.001*	*< 0.001*	*0.003*

Vibration perception thresholds are expressed in dB. Data are median [IQR]. P-values are based on Wilcoxon’s signed-rank test

Values in italics represent statistically significant differences

When we compared VPTs between the index finger and little finger in the hand with CTS, the VPTs were significantly higher in the index finger at 16, 250, and 500 Hz. However, they were significantly lower in the index finger at 32 and 64 Hz.

Thirty-eight patients had unilateral CTS, as confirmed by symptoms, clinical signs, and ENeG. In these patients, the VPTs in the index finger of the hand with CTS were significantly higher at all frequencies, than in the index finger of the healthy hand.

### Correlations

There were no correlations between the VPTs and touch threshold, SSS, or ENeG parameters.

## Discussion

This study showed that patients with CTS had significantly higher VPTs in the finger pulps of both the index finger and the little finger than healthy controls, at high as well as low frequencies. Furthermore, patients with unilateral CTS had significantly higher VPTs in the index finger of the hand with CTS than in the index finger of the healthy hand.

Electrodiagnostic studies are often used as reference standard for the diagnosis of CTS. However, these studies have false-positive and false-negative results, and the evidence for the role of electrodiagnostic tests in the diagnostic work-up of patients with suspected CTS is being questioned [17]. ENeG assesses function in the nerves from the basal phalanx of the fingers and proximal up into the forearm, whereas analysis of VPTs at different frequencies assesses function in both the afferent nerves and the peripheral receptors. Thus, compared to ENeG, a multi-frequency vibrometry can provide additional information that may be valuable in some patients.

Based on ENeG, which is thought to reflect the degree of compression of the median nerve, Padua et al. [18] classified CTS into six groups with mild and moderate being the two most common, constituting about 60% of patients. It has been suggested that multi frequency vibrometry can help in staging the degree of nerve compression [14]. However, we could not detect any difference in VPTs between mild and moderate CTS. The differences in ENeG parameters between mild and moderate CTS are subtle. We suggest that studies involving patients from all Padua stages should be done in order to determine whether analysis of VPTs at multiple frequencies can be used to stage the degree of compression of the median nerve in patients with CTS.

The present study corroborates previous results showing pathological nerve conduction [23] and increased VPTs also in the ulnar nerve-innervated little finger in patents with CTS [11]. The carpal tunnel and Guyon’s canal - where the ulnar nerve is located - are located next to each other in the wrist, and we speculate that the increased pressure in the carpal tunnel causing the CTS is also transferred to Guyon’s canal and the ulnar nerve. Another possibility for the pathological nerve conduction and increased VPTs in both the median and ulnar nerves is a pathological process in the forearm and wrist that results in neuropathy in both the median nerve and the ulnar nerve. CTS is known to result in structural as well as functional changes in the primary somatosensory cortex in the brain [24]. Thus, a third possible explanation, for reduced sensation in the little finger, is that these cerebral changes also effect neurons processing sensory information from the ulnar nerve.

A frequently quoted reason for not using ENeG in the diagnostic work-up for CTS is that there is no absolute correlation of results from nerve conduction studies with clinical symptoms. This was also the case in the present study, where there was no statistically significant correlation between subjective experience of symptoms assessed with the SSS and ENeG or VPTs in patients with mild and moderate CTS according to Padua. However, a correlation between neurophysiological severity, expressed on the seven point Canterbury scale, and surgical prognosis have been described [25]. Furthermore, costs, patient discomfort, and the need for specialized staff have all been advocated as reasons for not doing ENeG in patients who are suspected of having CTS. However, multi-frequency vibrometry also needs specialized staff and carries a cost, but there is no discomfort. An important difference between the two examinations is that multi-frequency vibrometry requires patient cooperation. In an ENeG examination, the patient has to endure the discomfort from the stimulation current, but patient cooperation is not needed for doing the examination. On the other hand, multi-frequency vibrometry is completely dependent on a cooperating patient who understands the examination, and who can remain focused during the procedure; thus, an unfocused patient and not a disorder in peripheral receptors or the peripheral nerve might cause a pathological VPT.

An alternative diagnostic tool for assessing patients with suspected CTS is neuromuscular ultrasound. Ultrasound of the median nerve has shown strong evidence for accuracy in diagnosing CTS [26] and it is completely painless, specific and sensitive and does not require patient cooperation however, it needs specialized staff.

Further studies are needed to determine the benefit of multi-frequency vibrometry, ENeG and ultrasound, both when they are used as sole diagnostic instrument but also when they are used in combination, for the diagnosis of CTS.

This study had some limitations. Although CTS is common, recruitment of patients was slow for three main reasons; 1. A large number of patients referred to the clinic due to suspected CTS did not fulfill the inclusion criteria of having a fractionated sensory nerve conduction velocity across the wrist of 40 m/s or less. 2. A number of patients were unable to manage the PROMs due to language problems. 3. Many patients had the impression that CTS can only be treated with prompt carpal tunnel release, and knew people who were satisfied with this procedure, so they did not want to participate in any studies that could delay their operation.

## Conclusions

The results of this study suggest that multi-frequency vibrometry could have a role in the diagnosis of CTS. However, we do not consider that this method would be applicable to clinical routine, as a diagnostic tool, at this stage. Further studies are needed to identify the role of multi-frequency vibrometry in the diagnostic work-up of patients with suspected CTS. Of special interest is the possibility of detecting pathology in the peripheral receptors of the fingers, as seen for example in patients with sensory disturbances due to long-term exposure to hand-held vibrating tools. These patients can have symptoms that mimic the symptoms seen in patients with CTS. It is also well-known that patients can have clinical symptoms of CTS, a normal ENeG, and can improve after median nerve decompression. It would be interesting to investigate whether such patients have normal VPTs, or whether a normal multi-frequency vibrometry result can rule out CTS.

## Abbreviations

CTS: Carpal tunnel syndrome
ENeG: Electroneurography
PROMs: Patient-rated outcome measures
SSS: Symptom severity score from the Boston carpal tunnel questionnaire
SWM: Semmes–Weinstein monofilament
VPT: Vibration perception threshold
